# Early perceptions of an epidemic

**DOI:** 10.1098/rstb.2008.0082

**Published:** 2008-11-27

**Authors:** Warwick H. Anderson

**Affiliations:** Department of History and Centre for Values, Ethics and the Law in Medicine, SOPHI, University of SydneyA14-Quadrangle, NSW 2006, Australia

**Keywords:** kuru, Fore, cannibalism

## Abstract

This article surveys some descriptions of the Fore people made on early contact in the 1950s by patrol officers, social anthropologists and medical doctors. Sorcery accusations and cannibalism initially impressed these outside observers, though gradually they came to realize that a strange and fatal condition called kuru was a major affliction of the Fore, especially women and children. Fore attributed kuru to sorcery, anthropologists speculated on psychosomatic causes and medical officers began to wonder if it was a mysterious encephalitis.

Although the Fore people must have heard of missionaries, prospectors, government officers and even anthropologists in the adjacent parts of the highlands, they had little direct experience of intruders until the 1950s. A few prospectors wandered through their land in the 1930s, and at least one plane crashed there during the war. New diseases such as influenza began to penetrate the high valleys in the vanguard of white contact. From the late 1940s, successive government patrols passed further into Fore territory (Nelson 1996). Patrol officers, or kiaps, would line up each hamlet and try to conduct a census, and seek out local ‘big men’ who might represent the colonial authorities as luluais (headmen) or tultuls (deputies). Usually, they lectured the villagers on the importance of hygiene and road construction and the need to give up warfare, sorcery and cannibalism. A police post was set up at Moke (later called Okapa) among the North Fore in 1951, with a kiap, John R. McArthur, stationed there from 1954 when the rough track from Kainantu opened. By 1957, it was possible to take a Land Rover or motorbike down to Purosa in the South Fore. It soon became clear to the colonial authorities that there were at least 12 000 Fore to know and control. Ronald and Catherine Berndt, Australian anthropologists, spent some time on the northern margins of the district in 1953. Missionaries and trade stores began to penetrate further south during this period. The smell of trade tobacco and kerosene mixed with the older highland odours of wood fires and pig fat, or ‘grease’. Within a few years, some northern villages were planting and harvesting coffee, and the economy had begun to monetarize. But only at the end of the decade did the scientific developments that so altered the lives of the Fore start to exert their influence.

In these post-war years, New Guinea became a magnet for young Australian men seeking adventure and anthropologists eager to make first contact with ‘primitive’ societies (Hasluck 1976; Downs 1980). By the end of the 1950s, even the previously isolated Fore people had come to know these types well. Their interactions with government officers and anthropologists, as with missionaries, traders and even dilatory medical doctors, became increasingly adroit and rewarding.

In his report on the patrol of the Fore region in May 1951, Gordon T. Linsley wrote unusually extensive ‘anthropological notes’ on the people he saw. Above all, the pace of social change astonished him. Although the patrol post at Moke had only recently opened, the surrounding area was already well controlled and village warfare had abated. Young Fore men seemed glad to find an excuse to give up fighting. Rest houses appeared in the larger villages, and some hamlets were moving down from the ridges and closer to the gardens. Separate men's houses persisted in only the more isolated settlements. Broad paths now linked communities, and many people regularly used the rough track to Kainantu, which allowed them to view new fashions and hear Pidgin. Beyond Moke, people no longer appeared nervous when the patrol approached. Close to Purosa, however, sorcery accusations abounded and fighting between districts continued.

Linsley anticipated other interpreters of the Fore in focusing on sorcery. The belief that illness and death were consequences of sorcery was ‘rampant’, prompting most of the distrust and fighting among villages in the region. Linsley despaired at ever convincing Fore that sorcery was a mere superstition.^1^ Although undoubtedly troubling, sorcery evidently offered them a coherent and convincing explanation of unexpected illness and death, and kiaps struggled to find a satisfactory substitute. The patrol officers' grasp of contemporary microbiology and theories of disease causation was rudimentary at best, and they lacked a ready means of translating the little scientific knowledge they possessed. Simple disparagement of sorcery was futile. Visiting the village of Yagusa in June 1952, W. J. Kelly saw small leaf-wrapped packages containing flesh of some kind, apparently used to detect the origins of sorcery.^2^ Later the same year, a cadet patrol officer heard that sorcerers took something intimately connected with the intended victim, such as hair, discarded food or faeces, then wrapped it with a stone in leaves, and placed the bundle on swampy ground. As the coverings decayed, so the victim weakened until death occurred.^3^

On patrols in 1953 and 1954, John McArthur identified this form of sorcery as ‘kuru’, a word that described the characteristic shivering and trembling of the victim, and distinguished it from the other magical operations. ‘Certainly it is difficult to reason out ‘kuru’’, he wrote. ‘It must surely be psychological’. He called together the villages around Moke and made a great bonfire of all materials that might be used in kuru. After that, he heard few sorcery accusations.^4^ But the following year, John Colman observed that sorcery was still the chief ‘impeding factor’ in development of the Fore. In one hamlet, the patrol officer watched the wife of a tultul ‘sitting dejectedly shaking violently all over’, a victim of kuru sorcery. ‘All this to the outsider is unbelievable and difficult to reason, but to the native it is a fearful shadow’.^5^

Late in 1952, Ronald and Catherine Berndt, a young anthropologist couple from Sydney, hiked south to Busarasa [Pusarasa] and Moke in Fore territory. They were ‘on the fringe of the restricted region, where inter-district feuding and cannibalism are still prevalent’.^6^ The people proved timid and the Berndts found conditions frustrating. Mist and rain swept across the mountains, and they spent most of the next few months shivering in the cold and attempting to dry themselves. Catherine admitted to finding the Fore ‘rather trying at times’. She lamented that they viewed the outsiders as a ‘source of supply and constantly expect us to restore to them as rightful owners the ‘cargo’ of which they had been cheated’.^7^ She was disappointed that the society was so materialistic and dominated by men. There was ‘plenty of cannibalism too’, she reported. ‘It's reasonably safe to visit some of the nearer villages, but the others are only for strongly armed patrols with plenty of native police. We haven't any illusions about these people’.^8^ Ronald wrote to Raymond Firth, his PhD supervisor at the London School of Economics:

Even where open fighting is no longer practised, there is always the alternative of sorcery (very popular in these parts, at least by repute). It is certainly an interesting area, despite the existence of some features which we, personally, find somewhat uncongenial.^9^

He told A. P. Elkin that the Fore were ‘difficult people to deal with, and we have had to be stricter here than we were in Aboriginal Australia’. Ronald slept with a loaded pistol under his pillow, and one day resorted to firing it in the air to disperse some villagers who pestered him.^10^

According to Ronald Berndt, sorcery and the payback for alleged sorcery represented the main arenas for male aggression among the Fore. It was, the anthropologist argued, both an instrument of crime and a means of enforcing conformity to certain social norms. At the same time as it might resolve conflicts, it also served to exacerbate them. ‘The use or threat of sorcery provokes fear’, Ronald wrote, ‘and an accepted local response is aggressive action’ (Berndt 1962, p. 209). The Fore considered sorcery the real or underlying cause of many serious illnesses and most deaths. As anything that had been in contact with a person might become an ingredient in sorcery, great care was taken in secreting any ‘leavings’ or discards. Ronald learned about a variety of local types of sorcery, including kuru. This primarily afflicted women and girls, making them shake uncontrollably and leading to partial paralysis and loss of muscular coordination (Berndt 1962, pp. 218–19). Although the Berndts never observed the final stages, they heard that death was inevitable. At the time, such clinical manifestations appeared compatible with a hysterical reaction, a psychosomatic response to stress. As late as 1959, Ronald still maintained that social or cultural events might have such ‘far-reaching effects on the human organism itself, even to the extent of interfering so drastically with it that it ceases to function’ (Berndt 1959, p. 25). All the same, Catherine recalled talking about kuru with Margaret Mead, whose former husband Reo Fortune was a friend of her uncle. Mead suggested they should get the doctors involved, but nothing came of this advice^11^ (Berndt, C. 1992; Berndt, R. M. 1992).

Early patrol reports implied that the health of the Fore was generally good, apart from some tropical ulcers, scabies and the yaws that disfigured so many of those greeting the outsiders. But kiaps were unlikely to see many invalids on a brief visit. In June 1952, however, W. J. Kelly reported that the health of the South Fore was ‘deplorable’ and people were ‘literally rotten with yaws and infected injuries’. He tried to send 15 of the worst cases to Kainantu for treatment, but they refused to travel through hostile territory. The only solution was to station a medical assistant in the area.^12^ In the following year, a native medical orderly was posted to Moke, and it was planned to send another further south to Purosa, but a medical patrol advised caution as ‘the natives…are just beginning to come under control’.^13^ In the meantime many did go willingly to Moke for injections against yaws. The kiap wondered if the availability of an effective cure had dispelled assumptions that the disease resulted from sorcery.^14^

The Berndts and a few patrol officers had noted the occurrence of kuru sorcery, but at first it did not appear to represent a major problem for the Fore or the government. The condition was rarer in the north around Moke and, in any case, most sufferers were hidden from strangers. Some kiaps, though, began to suspect that something was seriously wrong. In October 1953, W. J. Hibberd wondered why he saw so few young women in Fore villages, but he did not press the issue.^15^ John Colman was sufficiently concerned and puzzled by kuru to send a sufferer to Kainantu late in 1955, where the district medical officer, Vin Zigas, observed her. Zigas made a provisional diagnosis of ‘acute hysteria in an otherwise perfectly healthy woman’.^16^

In the middle of 1956, Frank Earl, an Australian medical assistant, accompanied Colman on patrol to the South Fore to investigate a suspected gonorrhoea outbreak. There was little venereal disease, but kuru was rife. At Yagusa they found five early ‘cases’ of the ‘disease’, then at Amusi they heard that thirteen women and eight children had recently died of kuru, and saw three comatose victims. The absence of young girls and women in the region was marked. ‘These people’, Earl wryly observed, ‘appear very prone to sorcery’. Further on, ‘a small female child aged eight years was carried to patrol suffering from kuru. This child died before examination could be carried out’, he reported. In early August at Kamira, Earl finally expressed his growing scepticism and irritation towards psychogenic explanations for the condition:

Ten cases of ‘kuru’ were brought to patrol today, all in the final stages of the disease, and it seems almost certainly to be encephalitis. Previous reports claim this disease to be psychological, but personally I am unable to see such effects on children under the age of two, if this were so, as I have seen today.

The impact of yaws treatment, and better knowledge of these strangers, may have encouraged Fore to disclose more kuru victims. But in this case, the medical officers could offer no injection, or ‘shoot’, to help sufferers. On 4th August, Earl was called back suddenly to Kainantu to care for his wife. He was ‘advised that Dr Zigas will be proceeding to investigate further’.^17^

In his unreliable memoir, *Laughing death*, Zigas claimed that John McArthur, the diffident kiap at Okapa, told him about kuru in September 1955 during a heavy drinking session in Goroka and invited him to see some sufferers (Zigas 1990). But there is no record of any visit to the Fore before October 1956, when he went to investigate Earl's suspicions. Accompanied by Liklik, a ‘very shrewd medical orderly’, Zigas managed over the following month to identify and study some 27 cases at Okapa and a further 11 in the neighbouring hamlets. Colman, whom he regarded as a ‘proud young man’ but an unusually thoughtful government officer, assisted him and even, according to Zigas, took him to the Kukukuku—though if so, it was not recorded in the patrol report (Zigas 1990, pp. 174, 163). Returning from Okapa with 22 samples of blood and a brain, he wondered what he should do next. He discussed the problem with Clarissa Andrea de Derka, the librarian at the Public Health Department, a stunning Hungarian blonde who presided over Port Moresby's only intellectual salon. She suggested that he send the materials to S. Gray Anderson, a virologist at the Walter and Eliza Hall Institute in Melbourne, Australia, who had recently investigated Murray Valley encephalitis near Moresby. She had also heard that F. Macfarlane Burnet, the Director of the Hall Institute, was interested in conducting further research in Papua and New Guinea. Zigas, imagining a new career as a clinical investigator, bypassed the government pathology laboratory in Moresby and posted the specimens directly to Melbourne.

Not until late December 1956 did Zigas write to John Gunther, the formidable Director of the Department of Public Health (Zigas 1990, p. 100). He reported that a number of people in the Okapa region, mostly women and children, were suffering from a new form of encephalitis, called kuru and attributed to sorcery. The disease began with lethargy, headache, vertigo and vomiting, progressing to tremors, unsteadiness, erratic eye movements, and then to loss of control of sphincters, inability to swallow and finally to death, usually within nine months of onset. The condition seemed limited to the Fore people. Zigas suspected that a virus caused the brain inflammation, and he speculated on the contribution of insects or birds to its transmission. A ‘large number of local influential natives’ approached him, begging him to ‘rid them of this killer’, just as medical treatment had recently eliminated yaws, which they ‘always thought was brought about by Hoodoo’. He concluded ambiguously: ‘They, in their primitive minds, now think that the Doctor has the power to beat their sorcerers’.^18^

Gunther immediately wrote a letter to Anderson and Burnet formally inviting them to become involved, and then asked Charles Julius, the methodical anthropologist in the Department of District Services and Native Affairs, to visit the region. In early 1957, Julius went to the area around Wanitabe and Kamira, further south than the Berndts had ventured 4 years previously. Julius detected only six different types of sorcery among the Fore, but the intensity of their obsession with kuru—‘for which lists of remembered dead were invariably long’—more than made up for such an impoverished repertoire. He recalled one of the kuru sufferers:

She was trembling spasmodically, and was not able to walk without assistance, her leg movements being jerky and uncontrolled. Her face seemed to have a rather fixed or ‘rigid’ appearance, and in speaking her words were slurred and indistinct. In discussion with her husband, he told me that she had been suffering from kuru for about two months, and that her condition was steadily becoming worse, although she was still being helped down to her garden every day.^19^

The woman's relatives had quickly settled on a known enemy as the culprit. They told the anthropologist how the sorcerer had obtained a piece of clothing and performed magical operations on it. In this case, divination rites had not been necessary, but Julius learned how they detected more elusive sorcerers too. In Wanitabe, people remembered 85 kuru deaths; in Amora, 72 deaths and in Okasa, 71 deaths. Most sufferers were women and children, and few if any seemed to recover. The Fore remained convinced that kuru, unlike some other forms of sorcery, was beyond the scope of European treatment. Julius advised Gunther that until a cure is discovered, ‘it seems improbable that any amount of explanation or propaganda will achieve much in removing what is the main cause of suspicion and insecurity in an otherwise unusually harmonious group’.^20^

The evident pragmatism and adaptability of the Fore during the 1950s made it hard for some outsiders to believe that they were dealing during this time with a devastating epidemic. In the circumstances, their dignity and fortitude, and their generosity to strangers, were remarkable. Soon, though, the Fore region would become for many condensed down simply to the ‘kuru region’.

## Endnotes

1 G. Linsley, Patrol Report Kainantu Sub-District, No. 3 of 1950/51, Central Highlands District, p. 16, Box 4, MSS 0215: Papua New Guinea Patrol Reports, Melanesian Studies Resource Center, Mandeville Special Collections Library, University of California at San Diego. Unless otherwise noted, all later patrol reports can be found here.

2 W. J. Kelly, Patrol Report Kainantu Sub-District, No. 8 of 1951/52, Eastern Highlands District.

3 R. R. Havilland, Patrol Report, No. 3 of 1952/53, Eastern Highlands District.

4 J. R. McArthur, Report of a Patrol Originating at Okapa, No. 4 of 1954/55, Eastern Highlands District, p. 9.

5 John Colman, Report of a Patrol of the South Fore Census Area, No. 14 of 1954/55, Eastern Highlands District, pp. 6, 7.

6 Ronald Berndt to Ralph Linton, April 3, 1953, 1952–53 Correspondence—New Guinea, Berndt papers, Berndt Museum of Anthropology, University of Western Australia.

7 Catherine Berndt to Phyllis Kaberry, [n.d.], 1952–53 Correspondence—New Guinea, Berndt papers.

8 Catherine Berndt to Janet Cotton, [1953], 1952–53 Correspondence—New Guinea, Berndt papers.

9 Ronald Berndt to Raymond Firth, [1953], 1952–53 Correspondence—New Guinea, Berndt papers.

10 Interview with Catherine Berndt, August 1992, Perth, Western Australia.

11 Catherine Berndt to Warwick Anderson, September 3, 1990, in author's possession.

12 W. J. Kelly, Patrol Report Kainantu Sub-District, No. 8 of 1951/52, Eastern Highlands District, pp. 7, 6.

13 G. H. Waldron, Forae-Kumano Linguistic Group, August 5–20, 1952, file 21/29/300: Patrol Reports, Eastern Highlands-Kainantu, box 3120, series 149, accession 23, Papua New Guinea National Archives, Pt Moresby, PNG.

14 John Colman, Report of a Patrol of the South Forei Census Area, 1956, Eastern Highlands District, p. 4.

15 W. J. Hibberd, Patrol Report, No. 6 of 1953/54, Eastern Highlands District, p. 4.

16 H. W. West to District Commissioner, Eastern Highlands, July 8, 1955, attached to John Colman, Report of a Patrol of the South Fore Census Area, No. 14 of 1954/55, Eastern Highlands District.

17 H. F. Earl, South Gimi, South Forei and Moke, June 21–August 5, 1956, file 21/29/300: Patrol Reports, Eastern Highlands-Kainantu, box 3120, series 149, accession 23, Papua New Guinea National Archives. See also John Colman, Report of a Patrol of the South Forei Census Area, 1956, Eastern Highlands District.

18 V. Zigas to Director of Public Health, December 25, 1956, Gunther papers, in the possession of Hank Nelson.

19 Charles Julius to Director of Public Health, February 28, 1957, pp. 2, 6, 8, Gunther papers.

20 Charles Julius to Director of Public Health, February 28, 1957, p. 9, Gunther papers.
